# Are modern contraceptives acceptable to people and where do they source them from across Nigeria?

**DOI:** 10.1186/1472-698X-13-7

**Published:** 2013-01-23

**Authors:** Obinna E Onwujekwe, Jane C Enemuoh, Chinwe Ogbonna, Chinyere Mbachu, Benjamin SC Uzochukwu, Agathe Lawson, Bannet Ndyanabangi

**Affiliations:** 1 Health Policy Research Group, Department of Pharmacology and Therapeutics, College of Medicine, University of Nigeria, Enugu, Nigeria; 2 Department of Health Administration and Management, College of Medicine, University of Nigeria, Enugu State, Nigeria; 3 Department of Community Medicine, College of Medicine, University of Nigeria, Enugu State, Nigeria; 4 United Nations Population Fund (UNFPA), New York, USA

**Keywords:** Modern contraceptives, Acceptability, Sources, Inequity, Nigeria

## Abstract

**Background:**

Understanding the extent that different modern contraceptives are acceptable to different populations groups and where they get the commodities from will help in developing specific interventions that will help to scale-up the availability of the contraceptives.

**Methods:**

The study took place in urban and rural sites in six states across Nigeria. Data on acceptability and sources of the contraceptives was collected from at least 770 randomly selected mostly female householders from each state respectively using a questionnaire. Acceptability of the different contraceptives was scored by the respondents on a scale of 1 (lowest) to 10 (highest). The relationships between acceptability and sources of the contraceptives with socio-economic status and geographic location of the respondents were examined.

**Results:**

The use of modern contraceptives in general was acceptable to 87% of the respondents. Male condom was the most acceptable means of contraceptive with an average score of 5.0. It was followed by implants with and oral contraceptive pill with average scores of 4.0, whilst IUD was the least acceptable with an average score of 2.9. The private sector was the major source of contraceptives to different population groups. Both male and female condoms were mostly procured from patent medicine dealers (PMD) and pharmacy shops. Intra Uterine Devices (IUDs) and implants were mostly sourced from public and private hospitals in the urban areas, whilst injectibles were mostly sourced from private hospitals. Oral contraceptives were mostly sourced from pharmacy shops and patent medicine dealers. There were SES and geographic differences for both acceptability and sources of the contraceptives. Also, the sources of different contraceptives depended on the type of the contraceptive.

**Conclusion:**

The different contraceptives were acceptable to the respondents and the major source of the contraceptives was the private sector. Hence, public-private partnership arrangements should be explored so that universal coverage with contraceptives could be easily achieved. Interventions should be developed to eliminate the inequities in both acceptability and sources of different contraceptives. The acceptability of all the contraceptives should be enhanced with relevant behaviour change communication interventions especially in areas with the lowest levels of acceptability.

## Background

Low level of modern contraceptive acceptability for sexually active men and women is a major factor that increases population growth in Nigeria [1-3]. A health sector target of the government of Nigeria is to meet the unmet family planning need in the country and keep the population growth within acceptable limits. The paper presents information on acceptability of modern contraceptives in the country. It also explores the different sources of the contraceptives.

Nigeria has an estimated unmet need for family planning at 20% and high total fertility rate (TFR) estimated to be 5.7 children per woman of reproductive age [4]. According to Nigerian National Demographic Health Survey of 2008, contraceptive prevalence rate was estimated to be 14.62% for any contraceptive method and 9.7% for modern contraceptives [5]. This translates to low level of use and is a major factor that contributes to unwanted pregnancy [4-6] and correlates to high maternal mortality ratios [5,7]. Also, 60% of unplanned pregnancies among women occur amongst those that are not using any form of modern contraceptives [3].

Hence, decision makers in area of family planning in Nigeria require information on the level of acceptability and sources of modern contraceptives amongst different population groups, so as to be able to make appropriate decisions for improved delivery and use of modern contraceptives in the country. There is an urgent need to achieve universal access to sexual reproductive health services in Nigeria, a target of the Millennium Development Goal 5, as well as the target of the National Strategic Health Development Plan and Vision 20: 2020 in Nigeria.

Modern contraceptives are in different forms: oral contraceptives pills, foaming tablets, injectibles, intrauterine devices (IUDs), implants, barrier method: condoms (male and female), and creams. Some of these are available in public or private clinic that offer family planning services of which most are administered by trained health providers (IUDs and implants) [4]. Usually most of the modern contraceptives that require insertion in the body or minor surgical operation for insertion are usually sourced from either government or privately owned hospital with trained health personnel, while other modern contraceptives that require few instructions on use are sourced from every other health provider [1,4]. This reflects some awareness by the consumers on where to source for contraceptives that require the services of trained health workers and those that do not.

Various forms of population characteristics, which have a bearing on equity in delivery of the contraceptives could either constrain or enable their equitable acceptability and access. Inequity exists when people are unjustly deprived of a need or something needed to protect oneself from unwanted condition [8]. It has been found that the lowest quintiles representing the poorest SES usually do not have equal access to life saving interventions and services unlike the highest (richest) quintiles although they desire the same good health as the rich [9]. Factors that could lead to barriers to access to modern contraceptive include cultural factors, religion, cost, side effect and availability of contraceptives which are probably related to level of acceptability of the different modern contraceptives [1,2,10]. Some studies suggest that the availability of new information about the current sources of contraceptives to consumers will help to improve the market for modern contraceptives [3,11]. However, it is not clear how differences in acceptability differ by type of modern contraceptive or the population group of the women.

The paper provides new information on the equity issues with regards to acceptability and sources of different contraceptives from different private and public channels across a huge country such as Nigeria. This is an area of study that existing literature have not explored fully, especially with a big sample size that covers different socio-demographic contexts in the country. Inequity in access to reproductive health services has always been a thorny issue despite the availability of various services [12]. The information generated by this study will help design policy measures to ensure that modern contraceptives are available and equitably accessible. Hence, the study provides knowledge that can be used to improve demand and delivery of modern contraceptives in Nigeria.

## Study methodology

### Study area

The study took place in the six-geopolitical regions of the country from August to October 2010. There are 36 states in Nigeria and a Federal capital (FCT) or Abuja, which is like a state. All the states and FCT are grouped into the six geopolitical zones and each geopolitical zone has minimum of five and a maximum of seven states. Six economically strategic states were purposively selected from each of the six geo-political zones (one state from each zone): North-central zone (FCT); North-west zone (Kano state); Southwest zone (Lagos state); Southeast zone (Enugu state); North-east zone (Adamawa state); and South-south zone (Rivers state). In each state, an urban and a rural area were selected for the study. Hence, there were 6 urban and 6 rural sites from the six states.

### Sampling and sample size

Adequate sample size was determined using a power of 80%, confidence level of 95% confidence level and utilization rate of contraceptives of 10%. This gave a minimum sample size of 350 per urban and rural site. However, in order to control for refusals and incomplete questionnaire, the number of respondents to be interviewed was increased to 385 per site, yielding at least 770 per state. This gave a total sample size of 4620 households for the entire study. In each state, the state capital and the most prominent rural local government area (LGA) were purposively selected. Then, the lists of political wards in each selected state capital and LGA were used to randomly select eight (8) wards in each state (4 urban and 4 rural) where the interviews were undertaken. Households were randomly selected from the sampling frame in the different study sites. The targeted respondent in a household was a female primary care giver of child bearing age (usually the wives), or in her absence, another female household members of child bearing age and in her absence after repeated visits, the male head of household.

### Study design and study tools

A pre-tested interviewer-administered questionnaire was used to collect information from the respondents on their level of acceptability and use of the major modern contraceptives. A pre-tested interviewer-administered questionnaire was used to collect information from the respondents on their level of acceptability and use of the major modern contraceptives. The questionnaire consisted of three different sections. The first was on the socio-demographic characteristics, the second was on the level of acceptability on the different modern contraceptives ranging from male and female condom, IUD, implant, injectibles, and oral contraceptives, and the third was the different sources where different modern contraceptives were accessed by respondents.

Trained enumerators with knowledge of English and respective local languages of the states and wards where they interviewed the people administered the questionnaire. Acceptability was elicited using two questions. The different contraceptives were explained to the respondents before they were asked the two questions to measure their level of acceptability. The first question was used to determine whether any of the contraceptives was acceptable to the people and the second question was used to score their level of acceptability of the different contraceptives (measured individually) on a score of 1 to 10, where 1 is lowest score and 10 is the highest score.

### Data analysis

Data on socio-demographic characteristics, acceptability and sources of different modern contraceptives that were elicited across the six urban and six rural areas were merged to yield an urban–rural data set. The dependent variables were levels of acceptability and sources of the different contraceptives. Inequalities in acceptability and sources of modern contraceptives were examined using an asset-based socio-economic status (SES) index that was created using principal components analysis from information on household ownership of radio set, bicycle, television set, motorcycle, fridge, as well as per capita weekly food value. The first principal component was used to derive weights for the SES index. The SES index was used to divide the households into (five groups) quintiles, which were: the least poor or the most well-off SES group (Q5), poor group (Q4); average group (Q3); very poor group (Q2) and the poorest group (Q1). The SES quintiles were used to compare the differences in level of acceptability and sources of the different contraceptives. Chi-square for trend analysis was used to determine the statistical significance of the relationship of the dependent variables with SES. In addition, the comparison of data set between the urban with rural areas was used to examine geographic differences in the dependent variables. The examination of SES and geographic inequities was based on chi-square tests and non parametric tests. The measure of SES inequity was the ratio of the lowest SES (Q1) and the highest SES (Q5).

Ethical approval was given by the Ethics Committee of the University of Nigeria Teaching Hospital, Enugu (UNTH). Written informed consent was obtained from the respondents before the interview commenced.

## Results

### Section 1: Socio-demographic distribution of the respondents in the six states

The response rate in the six states was more than 95%, although it varied slightly by state and a total of 4517 questionnaires were analysed. The numbers of analysable questionnaires ranged from 726 in Lagos state to 776 in Rivers state.

Table 1 shows that most of the respondents in the six states were either the wives or adult female household representative and were married. Most of the people also had some form of formal education and the commonest completed educational levels were senior secondary school (SSS), followed by universities or polytechnics and the average number of years that the respondents spent in school was 11 years. The average age of the respondents was 31 years. The number of household residents ranged from 4.9 in Lagos state to 8.3 in Kano state. The most common occupation of the people was petty trading/artisan, followed closely by unemployment.

**Table 1:** Socio-demographic distribution of the respondents in the six states

**Variable**	**Abuja N =771 n(%)**	**Adamawa N =728 n(%)**	**Enugu N = 769n(%)**	**Kano N = 747 n(%)**	**Lagos N = 726 n(%)**	**Rivers N = 776 n(%)**	**Combined N = 4517 n(%)**
Wife	405	664	658	460	537	584	3308
Adult female household rep	265	42	106	51	176	177	817
**Sex: Female**	671	708	763	509	711	761	4123
**Attended School**	742	460	730	581	710	762	3985
**Highest level of education**							
Primary School	54(7)	125(27)	152(21)	116(20)	60(8)	38(5)	545(14)
Junior Secondary School	40(5)	57(12)	66(9)	83(3)	23(3)	32(4)	301(8)
Senior Secondary school	264(36)	137(30)	275(38)	219(38)	299(42)	393(52)	1587(40)
Teacher training college	21(3)	8(2)	26(4)	31(4)	14(2)	16(2)	116(3)
College of Education	34(5)	71(15)	25(3)	47(8)	24(3)	82(11)	283(7)
University of Polytechnic	310	47	182	71	259	186	1055
Others	18(2)	14(3)	4(.5)	97(17)	28(4)	17(2)	178(5)
**Employment status**							
Unemployed	178(16)	321(29)	175(16)	155(14)	167(15)	95(9)	1091(25)
Subsistence farmer /herd keeper	19(3)	25(10)	74(2)	13(2)	1(.1)	53(7)	185(4)
Petty trader /artisan	136(18)	245(34)	237(31)	240(34)	93(29)	219(29)	1170(26)
Govt worker	127(17)	97(13)	99(13)	89(13)	25(3)	109(14)	546(12)
Private sector employee	90(12)	18(3)	64(8)	15(2)	48(7)	157(21)	392(9)
Big biz/self employed	158(21)	14(2)	95(12)	70(10)	327(45)	77(10)	741(17)
Others	77(10)	7(1)	39(5)	129(18)	58(8)	50(7)	360(8)
**Marital status:** Married	487(63)	652(90)	652(85)	664(94)	554(76)	589(77)	3598(81)
Muslim	186(24)	521(72)	5(7)	706(99.7)	148(20)	9(1)	1575(35)
Christian	586(76)	205(28)	762(99)	3(.4)	574(79)	751(98)	2830(65)
Traditional	2(.3)	1(.1)	0(.0)	0(.0)	2(.3)	2(.3)	7(.1)
Others	0(0)	1(.1)	0(0)	0(0)	0(0)	0(0)	1(.1)
**Age mean (SD)**	30.9 (8.0)	31.6 (7.6)	31.0 (7.1)	32.1 (7.0)	31.0 (6.7)	31.9 (6.2)	31.4 (7.1)

The distribution of the combined data from the six states by socio-economic status (SES) quintiles is shown in Table 2. The total numbers of cases in Q1 to Q5 were 904 (Q1), 904 (Q2), 903 (Q3), 903 (Q4) and 903 (Q5) and the total was 4517. Table 2 shows that Abuja and Rivers state had majority of people in Q4 and Q5, whilst Enugu and Kano states had the majority of people in Q1 and Q2.

**Table 2:** Distribution of socio-economic status (SES) quintiles by states

**States**	**Q1 (most poor) n(%)**	**Q2 (very poor) n(%)**	**Q3 (average) n(%)**	**Q4 (poor) n(%)**	**Q5 (least poor) n(%)**	**TOTAL n(%)**	**Chi-square (p-value)**
Abuja	62 (8.0)	121 (15.7)	110 (14.3)	195 (25.3)	283 (36.7)	771 (100)	(<.001)
Adamawa	160 (22.0)	185 (25.4)	149 (20.5)	133 (18.3)	101 (13.9)	728 (100)	(<.001)
Enugu	255 (33.2)	242 (31.5)	177 (23.0)	82 (10.7)	13 (1.7)	769 (100)	(<.001)
Kano	247 (33.1)	199 (26.6)	138 (18.5)	99 (13.3)	64 (8.6)	747 (100)	(<.001)
Lagos	113 (15.6)	88 (12.1)	209 (28.8)	162 (22.3)	154 (21.2)	726 (100)	(<.001)
Rivers	67 (8.6)	69 (8.9)	120 (15.5)	232 (29.9)	288 (37.1)	776 (100)	(<.001)
Total	904 (100)	904 (100)	903 (100)	903 (100)	903 (100)	4517 (100)	(<.001)

### Acceptability of different contraceptives in the six-geopolitical zones

The use of modern contraceptives was highly acceptable to the respondents (when asked whether the use of contraceptives was acceptable or not) and the combined data from the six zones showed that 87% stated that the contraceptives were acceptable. However, the proportions varied in the different states. The highest acceptability was found in Rivers state 757/776 (97.6%), followed by Abuja at 704/771 (91.3%). Others were: Enugu 698/768 (90.9%); Lagos 661/726 (91.1%). The least acceptability proportions were found in Adamawa state 599/728 (82.3%) and Kano 521/747 (69.7%).

On a score of 1 to 10 and using the combined data, male condom was the most acceptable means of contraceptive with an average score of 5.0. It was followed by implants with and oral contraceptive pill with average scores of 4.0, whilst IUD was the least acceptable with an average score of 2.9. The average score for female condom was 3.5, whilst it was 3.7 for injectibles. However, Table 3 shows that there were state by state statistically significant variations in the average acceptability scores for all the different contraceptives (p < 0.05).

**Table 3:** Average acceptability of different contraceptives in the six states

**Variable**	**Abuja Mean (SD)**	**Adamawa Mean (SD)**	**Enugu Mean (SD)**	**Kano Mean (SD)**	**Lagos Mean (SD)**	**Rivers Mean (SD)**	**Combined Mean (SD)**	***X*2 (p-value)**
Male condom	5.7(3.2)	3.2(3.8)	5.8(3.3)	1.8(2.6)	6.4(3.8)	6.9(2.5)	5.0(3.7)	1026.0 (<0.001)
Female condom	2.2(2.3)	1.8(2.4)	4.3(3.06)	1.0(1.3)	5.7(3.8)	6.1(2.9)	3.5(3.4)	1638.8 (<0.0001)
IUD	3.0(3.0)	1.7(2.5)	3.5(2.9)	1.0(1.4)	3.6(3.2)	4.5(2.8)	2.9(2.9)	1089.0 (<0.0001)
Implants	3.8(2.9)	4.1(4.2)	4.9(3.1)	1.9(2.7)	4.0(3.4)	5.3(2.6)	4.0(3.4)	1214.2 (<0.0001)
Injectables	3.2(2.9)	2.7(3.4)	4.9(3.2)	.9(1.1)	4.5(3.6)	5.5(2.5)	3.7(3.3)	614.1 (<0.0001)
Oral Contraceptive Pills (OCP)	4.3(3.1)	3.5(3.8)	5.1(3.3)	2.1(2.9)	3.5(3.2)	5.1(2.6)	4.0(3.3)	579.5 (<0.0001)

Tables 4 and 5 show that there were socio-economic status (SES) and geographic differences in acceptability of different modern contraceptives. In general, the acceptability scores for the different contraceptives increased as the SES quintile increased (p < 0.05). It was also found that modern contraceptives were generally more acceptable in the urban areas compared to the rural areas (p < 0.05). For instance, male condoms were the most acceptable contraceptive in the urban area with a score of 5.6, whilst the score was 4.9 in the rural area.

**Table 4:** Level of acceptability of different contraceptives by SES

**Variable**	**Q1 n(%)**	**Q2 n(%)**	**Q3 n(%)**	**Q4 n(%)**	**Q5 n(%)**	**Q1:Q5 ratio**	***X*2 (p-value)**
Male condom	3.9(3.8)	4.5(3.7)	5.3(3.7)	5.4(3.5)	5.8(3.4)	0.7	145.8 (.0001)
Female condom	2.9(3.2)	3.0(3.2)	3.8(3.5)	4.0(3.5)	3.9(3.3)	0.7	119.0 (.0001)
IUD	2.2(2.6)	2.4(2.6)	2.9(2.9)	3.2(3.0)	3.8(3.2)	0.6	202.1 (.0001)
Implant	3.1(3.4)	3.2(3.2)	3.8(3.3)	4.0(3.3)	4.1(3.2)	0.8	102.6 (.0001)
Injectible	3.5(3.5)	3.8(3.4)	4.2(3.4)	4.2(3.2)	4.3(3.2)	0.8	57.6 (.0001)
Oral Contraceptive Pill	3.5(3.5)	3.7(3.3)	4.0(3.3)	4.2(3.2)	4.4(3.2)	0.8	63.1 (.0001)

**Table 5:** Level of acceptability of different contraceptives by urban–rural location

**Variable**	**Urban n(%)**	**Rural n(%)**	**Urban:Rural ratio**	***X*2 (p-value)**
Male condom	5.1(3.6)	4.9(3.7)	1.0	9.4 (.002)
Female condom	3.7(3.3)	3.3(3.4)	1.1	27.1 (.0001)
IUD	3.1(3.0)	2.7(2.9)	1.1	27.1 (.0001)
Implant	3.7(3.3)	3.7(3.3)	1.0	2.4 (.12)
Injectible	4.2(3.4)	3.8(3.3)	1.1	18.5 (.0001)
Oral contraceptive Pill	4.0(3.3)	3.9(3.4)	1.0	4.4 (.037)

### Source of contraceptives

Table 6 shows from the combined data that both male and female condoms were mostly procured from patent medicine dealers (PMD) and pharmacy shops. IUDs and Implants were mostly sourced from public hospitals, whilst injectibles were mostly sourced from the private sector. Finally, oral contraceptives were mostly sourced from pharmacy shops and patent medicine dealers. Other sources of the contraceptives stated by respondents were maternity homes and Community Health Workers (CHWs).

**Table 6:** Sources from the combined data for sources of different contraceptives across the six states

	**Contraceptives**					
**Providers**	**Male condoms n(%)**	**Female condoms n(%)**	**Implants n(%)**	**Injectibles n(%)**	**Oral pills n(%)**	**IUDs n(%)**
Public hospitals	41(7.2%)	10(20%)	28(50.9%)	37(17.5%)	52(13.7%)	37(47.4%)
Private hospitals	22(3.9%)	4(8%)	8(14.6%)	54(25.6%)	21(5.4%)	14(18.0%)
Pharmacy	273(48.0%)	13(26%)	1(1.8%)	29(13.7%)	179(47.2%)	0(0)
PMVs	188(33.0%)	3(6%)	1(1.8%)	12(5.7%)	82(21.6%)	0(0)
PHC	21(3.7%)	2(4%)	1(1.8%)	29(13.7%)	10(2.6%)	15(19.2%)
Others	24(4.2)	18(36%)	16(29%)	50(23.7%)	35(9.3%)	12(15.4%)

### SES differences in major sources of the modern contraceptives

There were SES differences in the sources of the different contraceptives (Table 7). The higher SES (better-off) quintiles generally mostly procured their contraceptives from government and private hospitals and pharmacy shops, whilst the lower SES (worse-off) quintiles mostly procured their contraceptives from patent medicine dealers (PMDs). Table 7 shows that there are some individual SES differences in the sources of the different contraceptives). The denominator in the table is the total numbers of respondents belonging to each quintile and not just the total numbers of people that sourced the contraceptives from the different providers. For instance, across the SES quintiles, the sources of different providers for female condom, there was irregular increase or decrease from the least poor to the poorest SES groups. The percentages are dependent on the method mix within each quintile. IUD was sourced from few providers, with no statistically significantly difference across the SES quintiles.

**Table 7:** SES differences in major sources of different contraceptives

**Variable**	**Q1 n(%) n = 904**	**Q2n(%) n = 904**	**Q3n(%) n = 903**	**Q4n(%) n = 903**	**Q5n(%) n = 903**	**Q1:Q5 ratio**	***X*2 (p-value)**
**Male condom**							
Government hospital	5(0.6)	5(0.6)	11(1.2)	5(0.6)	15(1.7)	0.3	105(0.33)
Private hospital	2(0.2)	6(0.7)	2(0.2)	5(0.6)	7(0.8)	0.3	4.8(0.304)
PMD	37(4.5)	40(4.4)	45(5.0)	42(4.7)	24(2.7)	1.6	7.4(0.118)
Pharmacy shop	27(3.0)	49(5.4)	56(6.2)	67(7.4)	74(8.2)	0.4	25.9(0.000)
Others	9(1.0)	10(1.1)	13(1.4)	31(3.4)	37(4.1)	0.2	34.8(0.000)
**Female condom**							
Govt hospital	1(0.1)	1(0.1)	2(0.2)	4(0.4)	2(0.2)	0.5	3.0(0.556)
Private hospital	0	1(0.1)	1(0.1)	1(0.1)	1(0.1)	0	1.0(0.910)
PMD	0	0	1(0.1)	1(0.1)	1(0.1)	0	2.0(0.735)
Pharmacy shop	0	1(0.1)	4(0.4)	6(0.6)	2(0.2)	0	9.0(0.062)
Others	4(0.4)	5(0.6)	2(0.2)	0	2(0.2)	2	5.9(0.210)
**IUD**							
Govt hospital	7(0.8)	3(0.3)	9(1.0)	10(1.0)	8(0.9)	0.9	4.0(0.408)
Private hospital	2(0.2)	3(0.3)	0	5(0.6)	7(0.8)	0.3	8.6(0.071)
PMD	0	0	0	0	0	0	N/A
Pharmacy shop	0	0	0	0	0	0	N/A
Others	3(0.3)	2(0.2)	0	2(0.2)	3(0.3)	1	3.0(0.557)
**Implant**							
Govt hospital	3(0.3)	6(0.6)	4(0.4)	4(0.4)	11(1.2)	0.3	7.4(0.116)
Private hospital	0	0	2(0.2)	1(0.1)	5(0.5)	0	10.8(0.029)
PMD	0	0	0	0	1(0.1)	0	4.0(0.406)
Pharmacy shop	0	0	0	0	1(0.1)	0	4.0(0.406)
Others	3(0.3)	4(0.4)	1(0.1)	1(0.1)	4(0.4)	0.3	3.5(0.471)
**Injectible**							
Govt hospital	8(0.9)	13(1.4)	13(1.4)	7(0.8)	11(1.2)		
Private hospital	7(0.8)	8(0.9)	8(0.9)	13(1.4)	18(2.0)	0.4	8.2(0.086)
PMD	5(0.6)	1(0.1)	1(0.1)	3(0.3)	2(0.2)	2.5	4.7(0.322)
Pharmacy shop	11(1.2)	30(3.3)	27(3.0)	15(1.7)	14(1.6)	0.8	15.2(0.004)
Others	4(0.4)	0	3(0.3)	2(0.2)	1(0.1)	4	5.0(0.286)
**OCP**							
Govt hospital	8(0.9)	13(1.4)	13(1.4)	7(0.8)	11	0.7	3.0(0.552)
Private hospital	2(0.2)	5(0.6)	4(0.4)	2(0.2)	8(1.3)	0.3	5.9(0.204)
PMD	10(1.1)	17(1.9)	26(2.9)	15(1.7)	14(1.6)	0.7	8.8(0.067)
Pharmacy shop	18(2.0)	29(3.2)	40(4.4)	43(4.8)	49(5.4)	0.4	17.7(0.001)
Others	0(0)	4(0.4)	4(0.4)	2(0.2)	5(0.6)	0	5.5(0.253)

### Geographic differences in major sources of the modern contraceptives

There were geographic differences in the sources of the different contraceptives (Table 8). The denominator is the total numbers of respondents from urban and rural areas. In general, the urban dwellers generally mostly procured their contraceptives from government and private hospitals and pharmacy shops, whilst the rural dwellers mostly procured their contraceptives from patent medicine dealers. There were specific geographic differences in the sources of the different contraceptives.

**Table 8:** Geographic differences in major sources of different contraceptives

**Variable**	**Urban n(%) n =2204**	**Rural n(%) n = 2313**	**Urban : rural ratio**	***X*2 (p-value)**
**Male condom**				
Government hospital	30(1.4)	11(0.5)	2.7	9.8(0.002)
Private hospital	19(0.9)	3(0.1)	6.3	12.5(0.000)
PMD	73(3.3)	115(5.0)	0.6	7.8(0.005)
Pharmacy shop	159(7.2)	114(5.0)	1.4	10.4(0.001)
Others	52(2.4)	48(2.1)	1.1	0.4(0.517)
**Female condom**				
Govt hospital	7(0.3)	3(0.1)	2.3	1.8(0.179)
Private hospital	2(0.1)	2(0.1)	1	0.0(0.961)
PMD	1(0.0)	2(0.1)	0.5	0.3(0.592)
Pharmacy shop	10(0.5)	3(0.1)	3.3	4.1(0.042)
Others	9(0.4)	4(0.2)	2.3	2.2(0.140)
**IUD**				
Govt hospital	21(1.0)	16(0.7)	1.3	0.9(0.331)
Private hospital	12(0.5)	5(0.2)	2.4	3.2(0.072)
PMD	0	0	0	N/A
Pharmacy shop	0	0	0	N/A
Others	7(0.3)	3(0.1)	2.3	1.8(0.174)
**Implant**				
Govt hospital	17(0.8)	11(0.5)	1.5	1.6(0.206)
Private hospital	6(0.3)	2(0.1)	3.0	2.2(0.138)
PMD	0	1(0.0)	0	1.0(0.329)
Pharmacy shop	0	1(0.0)	0	1.0(0.329)
Others	9(0.4)	4(0.2)	2.3	2.3(0.140)
**Injectable**				
Govt hospital	64(2.9)	33(1.4)	1.9	11.7(0.001)
Private hospital	38(1.7)	16(0.7)	2.4	10.1(0.001)
PMD	1(0.0)	11(0.5)	0.1	7.9(0.005)
Pharmacy shop	19(0.9)	10(0.4)	1.9	3.3(0.071)
Others	1(0.0)	9(0.4)	0.1	6.0(0.014)
**OCP**				
Govt hospital	26	26	1	0.031(0.861)
Private hospital	15(0.7)	6(0.3)	2.5	4.3(0.038)
PMD	27(1.2)	55(2.4)	0.5	8.4(0.004)
Pharmacy shop	102(4.6)	77(3.3)	1.3	5.0(0.25)
Others	12(0.5)	3(0.1)	4	5.9(0.0150

## Discussion

The different modern contraceptives were largely acceptable to the respondents, although the level of acceptability varied across the states, with the lowest levels of acceptability recorded in the two northern states of Kano and Adamawa states. The fact that the lowest levels were recorded in these two states is potentially related to the fact that the North had lower contraceptive prevalence rate than the South [13]. This could be as a consequence of cultural, religious and educational factors. Some authors found that religion played a major role in acceptance of modern contraceptives in northern Nigeria [14].

Low level acceptability of the modern contraceptives in some states leads to women unmet needs and it also traps families into poverty and leads to diseases associated with maternal health [15]. These points to the need to increase awareness about contraceptives especially in the northern states so as to demystify contraception and enable people make good contraceptive choices. The higher acceptability in the urban areas compared to the rural areas reflects the higher educational status of urban dwellers on acceptability of the contraceptives. The general high level of acceptability implies that if the contraceptives are readily available in both the public and private sectors and are affordable, the level of use of the contraceptives will increase.

It was interesting to find out that most of the contraceptives were from private sector, especially the private drug retailers (patent medicine vendors (PMDs) and pharmacy shops. Several studies that were undertaken in Nigeria, Ghana and Kenya have also shown that although some people source their contraceptives from the public sector, the contraceptives are mostly sourced from the private sector such as the PMDs, pharmacies and private hospitals [16-19]. Other previous studies also found that some contraceptives were mainly obtained from the public sector in Nigeria [20]. However, a study found that public facilities were the major source for contraceptives and that the government played a major role in the provision of the services [21]. The implication is that the importance of the private sector should be recognised by policy makers and that private sector should be incorporated in the formal deployment of modern contraceptives for ensuring widespread availability of the commodities.

It was apparent that the level of proximity of the different providers to consumers had an effect on peoples’ sources of contraceptives. Patent medicine dealers and pharmacy shops being the most readily accessible providers were the major sources of the contraceptives in this study. It could be argued that providers located far from users could result to poor access and usage. However, the differential level of use from different providers could also be a result of relative availability of different contraceptives in different providers and privacy people get while accessing different contraceptives as was found by a study done in Kenya increased use of the private sector was due to their proximal location to the consumers and confidentiality and privacy of information given to the patient [19]. However, the finding on the sources of different contraceptives was insightful. It appeared that consumers were knowledgeable enough to patronise hospitals for the higher tech contraceptives (IUDs, Injectibles and Implant), whilst they visited informal private health providers and drug retailers for condoms and OCP. The finding should be used to inform programmatic designs on the involvement of the private retailers in official interventions to scale-up the availability of contraceptives. The capacity of the drug retailers should be developed further, especially in the information that they provide the consumers since some studies have found that a significant amount of information given by some these health providers could be incorrect [21].

There were some levels of inequities in acceptability and sources of the contraceptives. The least poor (highest) SES groups found it easier to source or access contraceptives from different providers than the poorer SES groups. Other studies also found inequities in access to modern contraceptives by different SES groups [22]. Some studies argue that people in the poorer SES may not be aware of policies designed to increase their access to reproductive services (such as the contraceptives) [23]. Strategies to promote equity in acceptability and access to the contraceptives should include the use of innovative health education campaigns at the community to create the awareness of the existence of the national policy on abolition of user fees for modern contraceptives in the public sector in Nigeria. Some authors argued that women in the lowest SES have the highest level of unmet needs and are least likely to access, spend on and use modern contraceptives [24].

There were also SES and geographic inequities in the sources of the different contraceptives, with the better-off quintiles generally mostly procured their contraceptives from government and private hospitals and pharmacy shops, whilst the worse-off quintiles mostly procured their contraceptives from patent medicine dealers. This could be a function of both financial and geographic access. Nonetheless, it was argued that despite the interest in determining socio-economic inequality in access to modern contraceptives, only few factors associated with it are known [8].

However, the study has some limitations and a major one was the fact that the questionnaire was in English language. However, the interviewers were encouraged to use the local languages to explain some of the points and ask some of the questions to the respondents. This was possible since the interviewers were recruited from the respective states so as to help to eliminate language barriers. The lack of a supportive qualitative study that would have been used to deeply probe the reasons for the different levels of the acceptability of the contraceptives in the different geographic settings is another limitation of the study. Qualitative studies should be undertaken in the future so as to gain better understanding of the factors behind the levels of acceptability and sources of different contraceptives in different contexts and states. Also, detailed work on potential role of the public and private sectors on improving equity of access to modern contraceptives should be the subject of future studies.

## Conclusions

Overall, the private sector, especially patent medicine dealers and pharmacy shops should be made part of interventions to scale-up deployment and use of contraceptives, as they are currently very major source of the goods. However, interventions should be instituted so that the public sector also becomes a major source of contraceptives. The deployment of contraceptives in the public sector, especially at the primary healthcare (PHC) level should also be scaled-up with necessary investments by government in sustainable provision of free family services. This is because PHC centers are widely located in both the urban and rural parts of Nigeria. Deployment of these contraceptives at the PHC will improve equity in the accessibility and useamong the women in the community especially for implants and IUDs because women are more likely to use the modern method of contraceptives when they know where the services are available [5]. However, there should be public private partnerships to improve equity in access to all modern contraceptives and build up the quality of services delivered by the private sector since they are currently the most common source of modern contraceptives in Nigeria, whilst enhancing the services in the public sector.

## Competing interests

The authors declare that they have no competing interests.

## Authors’ contributions

OO, CO, AL and BN conceived the study. OO and CO participated in development of data collection tools and data collection. OO and JCE performed statistical analysis. JCE, CM and BSCU participated in the design of the study, data collection and analysis. OO, JCE and CM drafted the manuscript. All authors read and approved the final manuscript

## Pre-publication history

The pre-publication history for this paper can be accessed here:

http://www.biomedcentral.com/1472-698X/13/7/prepub
